# Caring for Transgender People in Healthcare: A Qualitative Study with Hospital Staff in Croatia

**DOI:** 10.3390/ijerph192416529

**Published:** 2022-12-09

**Authors:** Ivana Tutić Grokša, Robert Doričić, Vanja Branica, Amir Muzur

**Affiliations:** 1 Department of Social Sciences and Medical Humanities, Faculty of Medicine, University of Rijeka, 51000 Rijeka, Croatia; 2 Department of Public Health, Faculty of Health Studies, University of Rijeka, 51000 Rijeka, Croatia; 3 Department of Social Work, Faculty of Law, University of Zagreb, Nazorova 51, 10000 Zagreb, Croatia

**Keywords:** transgender people, hospital staff, healthcare managers, hospital team members, healthcare system, Croatia, qualitative research

## Abstract

Transgender and gender-diverse people have greater health risks due to increased social stress and face a disadvantaged position in the healthcare system as a result of the stigma associated with their gender identity. Due to the lack of research in Croatia on the position of transgender people in the healthcare system, this research was intended to supplement the knowledge about the experiences of hospital staff in the Croatian healthcare system when caring for patients with transgender identities. Qualitative research was conducted using an interview method. The participants (*n* = 10) were healthcare managers or hospital care team members. The collected data were processed through thematic analysis. The results show that some participants had had no encounters with transgender patients and those who had described them as unproblematic or had only encountered them at a level of basic healthcare. They also described how they perceive transgender people and their life circumstances. The participants described how they envision potential encounters with this group of patients and what they consider necessary to improve the position of this group within the healthcare system. In the discussion part of the article, we assess the need for additional training regarding hospital staff, especially in terms of diversity competence, and for an increase in the visibility of transgender patients.

## 1. Introduction

Transgender and gender-diverse people are those whose gender identity or expression does not conform to the gender socially associated with the sex assigned to them at birth [1] (p. S5). Some authors observe transgender and gender-diverse identities as a reflection of the multidimensionality of gender, that is, a “departure” from the traditional binary conception of gender [2,3]. Throughout the paper, the terms “transgender people” or “patients with transgender identities” will be used, as these were mostly used during the conduct of the research presented in this paper. However, these terms do not exclude people with other gender minority identities.

The gender minority stress model recognizes that transgender people experience increased stress due to discrimination, violence, and rejection connected with gender identity/expression or nonacceptance of gender identity [4,5]. Because of such stressors, transgender people are at increased risk of adverse health outcomes [4]. The discrimination, violence, and rejection experienced by transgender people are a reflection of the stigma they face. Stigma occurs due to traditional norms that promote and recognize exclusively cisgender (those whose gender identity matches the sex assigned to them at birth) and heterosexual people, placing those who “deviate” from these norms in a socially disadvantaged position [6]. Transgender stigma can be present in structural and interpersonal forms [7]. Some of the forms of structural stigma are stigmatizing policies and practices, a lack of training of public service providers, or barriers when accessing healthcare, while interpersonal stigma is present in, for example, the discrimination in the healthcare system and in the workplace, rejection by family, or hate crimes.

There are various barriers that make access to the healthcare system difficult for transgender people. These can be divided into structural, interpersonal, and anticipation barriers [8]. Structural barriers are present at the level of the entire system (e.g., limited access to transgender care); the interpersonal barriers relate to the behavior of healthcare professionals and their lack of knowledge or skills, and the anticipation barriers include the reluctance of patients to seek healthcare due to expectations related to the previously described types of barriers [8]. In other words, obstacles include direct discriminatory behaviors, such as verbal violence, but also indirect discrimination, such as an insufficient adaptation of the system to the needs of patients with transgender identities [9]. What is also common for transgender people, that is, members of sexual and gender minorities in general, is that when accessing the healthcare system, they are invisible to healthcare providers [6,10].

Previous research in Europe shows that the experiences of people with transgender and gender-diverse identities within the healthcare system are varied [11,12,13,14]. Research by the European Union Agency for Fundamental Rights, conducted within the countries of the European Union, showed that 54% of transgender people included in the research had experienced discrimination, violence, or harassment because of their gender identity [11]. Such events took place during employment or in the workplace, or in the education, healthcare, or social welfare systems. As far as healthcare services are concerned, out of the 39% of respondents who sought medical/psychological help, 71% reported positive experiences with psychologists, psychiatrists, or other specialists, and 45% reported positive experiences with a family medical doctor. On the contrary, research in Austria has shown that the behaviors of healthcare professionals can be negative and include respondents occasionally or frequently “not being taken seriously”, misgendering (even after highlighting a preferred name/personal pronouns), verbal abuse, and even refusal to provide healthcare [12]. This research also shows that gender nonbinary individuals have a worse perception of relationships with medical doctors than transgender individuals. Other research conducted in Sweden indicates that transmasculine people feel safe when seeking healthcare if their gender identity is accepted and normalized, while the pathologizing of gender identity and mistrust leads to negative emotions [13]. “Exposed situations” (e.g., gynecological examinations) are especially important in the perception of healthcare, as transmasculine people are extremely in need of respect, openness, and empathy. Another Swedish study shows that, although the participants have positive experiences, they also encounter ignorance and a lack of knowledge, discomfort, rudeness, or disrespect from healthcare professionals, along with unwanted disclosure of their gender identity in front of other people, and so on [14].

Compared to research on the experiences of healthcare from the perspective of transgender people, there is a smaller number of studies conducted in European countries on samples of healthcare professionals related to their experiences of caring for transgender people [15] (p. 2). One of these studies is a qualitative study of the attitudes and experiences of healthcare professionals (*n* = 13) when caring for transgender men undergoing fertility preservation by egg freezing [15]. Participants spoke about the challenges of maintaining professionalism, stating that it was necessary to learn the needs of a new group of patients while developing their professional skills and designing new ways to provide healthcare. The challenge for their professionalism was confronting preconceived notions and assumptions based on cisnormative values.

As expected, transgender people are in a disadvantaged position in many areas of Croatian society [16]. The perception of the general population is reflected in the data collected during the research conducted by the European Commission [17]. For example, only 39% of respondents from Croatia support changing gender markers in legal documents. If they had a colleague at work with transgender identity, 39% of respondents said they would feel completely comfortable, while only 18% would feel comfortable if their child’s partner was a transgender person [17]. Members of gender minorities/transgender people are also recognized by the Office for Human Rights and the Rights of National Minorities of the Croatian government as one of the groups whose health and access to the healthcare system are unfavorable [18]. This disadvantaged position is reflected in healthcare services that are insufficiently adapted to the specific needs of transgender individuals and health professionals who are not adequately sensitized to these needs [18] (pp. 25–26). Likewise, both nongovernmental organizations [19,20] and the Ombudsperson for Gender Equality [21,22,23,24] recognize that access to healthcare for transgender people in Croatia is inadequate. Unfortunately, apart from information on healthcare services related to gender reassignment procedures, there is no information on the use of universal healthcare services by transgender people in Croatia.

According to the standards of the Council of Europe, the legal recognition of gender in a Member State of the European Union should be fast, transparent, accessible, and based on the principle of self-determination, but the current process in Croatia does not follow these standards [19]. In particular, the following issues are highlighted in the reports of the Ombudswoman for Gender Equality: the lengthy process to obtain a National Health Council opinion on gender reassignment, the reissuance of identity documents, including new personal documents after a medical procedure, and unregulated therapeutic and healthcare services [21,22,23,24]. Another issue is specifying the costs associated with gender reassignment that should be covered by mandatory insurance. Transgender individuals who have received favorable evaluations from psychologists and psychiatrists are eligible for hormonal therapy [19] (p. 76), and the Croatian Health Insurance Institute covers the costs of this and provides the necessary psychological assistance [20]. However, gender reassignment surgery is viewed as an aesthetic treatment and is not covered by the costs of mandatory insurance. Although there are reports about different practices in the past regarding payment for such surgery through mandatory health insurance [24], it is not systematically covered.

In Croatia, so far, a small number of studies have been conducted on access to healthcare for transgender people or their experiences in the healthcare system [25,26,27,28]. In research from 2014 on the experiences of people who had undergone medical gender reassignment, the participants talked about experiences of unpleasant and discriminatory behavior by health professionals, such as deprecation, a lack of understanding, and an unwillingness to provide medical help, along with being insulted, ignored, and provoked [25]. In another study from 2017, which was focused on the process of gender reassignment and the obstacles encountered during this process, the participants mentioned the medicalization and pathologization of transgender and gender-diverse identities and the questionability of the sensitization and training of experts involved in the procedure [26]. In research on access to healthcare for transgender people during the COVID-19 pandemic conducted in 63 countries in 2021 (including Croatia), 61.2% of respondents had limited access to transgender-specific healthcare services during that period [27]. Barriers were more often experienced by people assigned as male at birth and those with lower incomes. In another study from 2021, internal documents of public healthcare institutions were analyzed to determine the regulation of minority groups’ access to the healthcare system [28]. None of the included health institutions had an internal document focusing on the specific needs of gender minority patients.

The World Professional Association for Transgender Health (WPATH) “envisions a world wherein people of all gender identities and gender expressions have access to evidence-based healthcare, social services, justice, and equality” [1] (p. S5). By starting with this vision, the purpose of our research is to expand knowledge about the current situation in the healthcare system in the Republic of Croatia to gradually improve the status of transgender people as patients. We consider our research important precisely because we focus on the perspective of healthcare employees. Learning about their experiences and attitudes will contribute to the improvement of patients’ positions in the long-term.

The aim of our research was to gain insight into how hospital staff in the Croatian healthcare system perceive the provision of healthcare services to patients with transgender identities. The research questions arising from the stated goal were the following:

(1) How do healthcare managers and hospital team members describe their experiences with patients with transgender identities?
(2) How do healthcare managers and hospital team members imagine their potential experiences with patients with transgender identities?

## 2. Materials and Methods

### 2.1. Research Design and Data Analysis

In order to achieve the aim of our research, a qualitative approach was chosen. The reasons for choosing this approach included a lack of previous knowledge about the researched topic and the need to hear the perspective of hospital staff. The research was conducted as part of the research project “Healthcare as a Public Space: Social Integration and Social Diversity in the Context of Access to Healthcare in Europe”. The qualitative research in the fourth phase of this research project was focused on the perception of access to the healthcare system from the perspective of patients with minority (national, religious, sexual, and gender) identities and hospital staff. The following research phases were carried out: the creation of the research design and materials; contact with “gatekeepers”; contact with participants; collection of data through interviews; transcription and analysis of the collected data; reporting results.

A purposive sample was used. The inclusion criterion was employment in the public healthcare system at the secondary and tertiary healthcare levels (specialist-consultative and hospital activities) that provide care to all patients, not only transgender patients. More specifically, we included healthcare managers and hospital care team members [29] in our research. Potential participants were healthcare managers and hospital staff who had been working with members of minority groups (which also included gender minorities). Following on from the research project mentioned above, it was estimated that data saturation would be achieved with 10 participants. Healthcare institutions were the gatekeepers. Therefore, their management forwarded the information about the research to their employees and, in turn, suggested participants assessed as being good informants to us. Initial contact with management (the directorate of hospitals or the assistant directors for quality) was made by e-mail. The e-mail contained information about the research, e.g., the aim, research methodology, ethical aspects, and how to contact the researchers, as well as the inclusion criteria.

The chosen method of data collection was a semistructured interview. Although this type of interview uses a series of predesigned questions, the open-ended type of questions allows for the exploration of individual experiences [30]. A short interview guide was designed (Appendix A). Before the interview, the participants were asked to sign an informed consent form. Out of 10 interviews, 8 were conducted face-to-face and 2 online. There was no follow-up interview. The average duration of the interviews was around 38 min, with the shortest being around 21 min and the longest around 57 min. Only part of the interview, lasting a few minutes, was focused on the goal of this research. Of the total number of participants (*n* = 10), seven were female, and three were male. All have a university degree or junior college education. Four participants are medical doctors, two are nurses, and the other four participants are psychologists, social workers, or economists by profession. The participant’s position within the healthcare institution is mainly an assistant director for quality. They are employed in different secondary and tertiary level healthcare institutions (general hospitals, clinical hospital centers) and live and work in different cities from all regions of Croatia. The interviews were conducted from October 2021 to January 2022. The interviews were audio-recorded and then transcribed using Sonix, an automated transcription software, and corrected by an external collaborator (an ethnologist and cultural anthropologist by profession), who signed a statement of confidentiality. The external collaborator was familiar with the transcription process and was given instructions for editing the obtained text records.

### 2.2. Data Analysis

Thematic analysis [31] was used for the data analysis. The first author conducted the initial analysis, while the other authors had the role of “neutral coders” to ensure the credibility of the data. Inductive coding was applied. The unit of analysis was a thought, part of a sentence, or one or more sentences. Data were coded and classified into themes “manually”, without the use of software.

**Figure 1:**
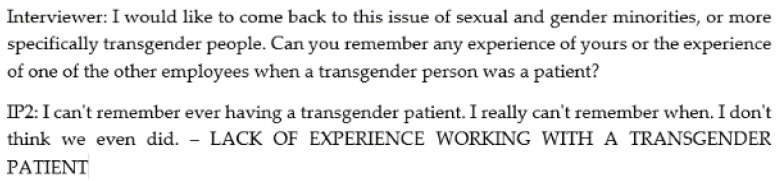
Example of coding.

The analysis included the following steps: (1) familiarization with the material; (2) extraction and coding of the parts of the transcripts; (3) classification of codes into themes; (4) repeated revising and naming of the themes; (5) interpretation of the results. Firstly, the transcripts were read in detail, and the parts of the text relevant to the research aim/research questions were marked. Then these parts of the text were coded, i.e., assigned a meaning (an example of coding can be found in Figure 1). In the Section 3 “Results”, the statements of the participants are presented to gain insight into the naming of the codes. Finally, the codes were grouped into subthemes and themes according to similarity. During the collection and analysis of the data, we estimated that data saturation had been achieved within the sample.

### 2.3. Ethical Aspects of the Research

This research sought to respect the principles of confidentiality, autonomy, well-being, and nonharm. Ethical approval of the research was provided through the Ethics Committee for Biomedical Research at the University of Rijeka, Faculty of Medicine. Certain healthcare institutions requested an additional application to their own ethics committee. Participants were informed in advance about aspects of the research, including the ethical aspects, through the Notice for Participants. All participants were of legal age and had legal capacity, so they could independently and thoughtfully decide on their participation. All participants signed a printed or electronic informed consent form. The transcription was carried out by an external collaborator who was asked to sign a confidentiality statement. To ensure confidentiality, all data that can be associated with a particular participant (e.g., personal names, names of cities and institutions, etc.) were deleted and replaced with symbols, such as “XX”. In addition, each participant received an identification mark, e.g., IP1 (Interview Participant 1). During the analysis phase of the research, the data were analyzed collectively.

### 2.4. The Research Team and Reflexivity

The authors practice within the fields of medicine, public health, and social work, which provided an interdisciplinary approach to all phases of the research. All authors are cisgender people, i.e., they do not belong to a gender minority, which is why it was necessary to be reflexive toward the methodological choices. All the authors have some previous experience with the topic of the position of gender minorities in public systems. The first and second authors of this paper conducted the interviews; both have previous experience with this research method. The researchers kept field notes to be able to refer back to regarding potential interfering factors. All the authors also have previous experience with the use of thematic analysis, which was used for the data analysis in this research. Any methodological dilemmas that occurred during the research were discussed among the research team members.

## 3. Results

In the collected data, four themes emerged: (a) experiences with the provision of healthcare regarding transgender patients; (b) the perception of the patients with transgender identities; (c) the assumed reactions of hospital staff to patients with transgender identities; (d) recommendations for improving the healthcare of patients with transgender identities. In the following subsections, the themes and subthemes will be presented and illustrated with participants’ quotes. The results should be seen in light of the fact that the participants were not involved in healthcare services specifically aimed at transgender people (e.g., gender reassignment procedures), but services for the general population.

### 3.1. Experiences with the Provision of Healthcare to Transgender Patients

The first presented theme summarizes participants’ experiences of healthcare being provided to transgender patients. Table 1. shows the subthemes related to this theme.

Most of the participants stated that they have no experience providing healthcare services to transgender patients (*I can’t remember ever having a transgender patient. I really can’t remember when. I don’t think we even did.*—IP2). Some mentioned that they generally do not discuss patients’ gender identity with their colleagues, suggesting the possibility that they may have encountered a patient with a transgender identity without being aware of it (*In the end, respect for the right to privacy and… in a way, the protection of personal data, these things are not even commented on at such a level in a way that we would prattle ‘Huh, there were two transgender patients.*—IP3).

Those participants who had been in contact with transgender patients said that their experiences so far had been positive and not specific in any way and that they had behaved in the same way as with other patients *(So, when I was working as an intern, I had several experiences with people who had changed their gender… There were no problems then…* —IP6). One participant pointed out that transgender patients in their hospital could access only certain healthcare services (“basic medical treatment”). These patients are referred to other institutions for more demanding specialist examinations, especially those related to gender reassignment procedures (*Availability of healthcare in the sense that if something like this happened, the urologist or gynecologist would recommend some treatment… all the basic equipment is here, but everything that the procedure requires,* i.e., *a psychologist and everything else and the procedure itself, is referred to higher centers*.—IP1).

The majority of the participants emphasized that healthcare provision is equal regardless of the gender identity of the patients, meaning that there is no discrimination against patients with transgender identity and that, therefore, the healthcare system is accessible (*I claim that there is no discrimination in access to healthcare or the provision of healthcare and care for people… in terms of gender identity and transgender people.*—IP6; *Does a cisgender person have a knee? Does a transgender person have a knee? Can his knee hurt?… So, his knee can hurt. So, there is no difference.*—IP9).

### 3.2. Perception of Patients with Transgender Identities

Another theme that emerged from the data relates to the perception of the characteristics of patients with transgender identities by healthcare managers and hospital team members. Table 2 shows the subthemes related to this theme.

The first subtheme shows how participants who had experience with transgender patients perceive them. Healthcare team members stated that transgender patients expect condemnation from others based on their previous experiences in their families and society. Healthcare team members describe patients with transgender identity as shy and withdrawn because of this expectation. In addition, shame that is connected with sexual health could be a factor in avoiding the seeking of medical help. As one participant pointed out, *they limit themselves in terms of healthcare because of shame. Therefore, it is very difficult for them to have any examination that includes painful places related to sexuality, because it means a direct questioning of their identity, experiencing incredulous looks… They have to take hormones every month. So, it’s all a process that lasts for years and it’s actually much more what they expect and maybe what they encountered somewhere along the way in their social environment and that’s what they expect in terms of health… I know some transgender people. Whenever I ask how it was, this and that, he says ‘Please don’t send me to the gynecologist.’ That’s the part when he says, ‘Everything else went well.’*—(IP5). Healthcare team members that are focused on mental health and who have had experience with patients with transgender identity talked about the increased possibility of mental disorders, meaning depression and substance abuse (*So, transgender people somehow have the most open door to severe depressive episodes. I’m talking about clinical depression and pills. They are more likely to consume psychoactive substances and drugs. So, it’s an escape from reality*.—IP5).

The second subtheme includes the perception of problematic interpersonal (family) relationships and the impact they have on the identity-building process and self-image (*These are always questions related to some kind of identity, acceptance of oneself as number one, but I think that there is a lot of condemnation from the family involved. This is what they heard from their parents*.—IP5). The only obstacle within the healthcare system that was described by healthcare team members is related to gender reassignment procedures. Participants who have experience with patients with transgender identity have knowledge of the rights that transgender people have based on health insurance, and the problems they face regarding, for example, a mastectomy that is not covered by an insurance policy (*There are some legal obstacles because I know that they are now related to a mastectomy, which is no longer paid for by health insurance*.—IP5).

### 3.3. Assumed Reactions of Hospital Staff to Patients with Transgender Identities

This theme summarizes participants’ assumptions about what an encounter with transgender patients would look like. Table 3. shows the subthemes related to this theme.

The first subtheme, “’Affirmative reactions”, includes a presumption of equal healthcare provision in the case of an encounter with a patient with a transgender identity *(If I were a transgender person, I would be treated the same as anyone else who is not, who is a man, who is a woman.*—IP3). The participants also pointed out their openness and acceptance of a “diversity” of patients (*Honestly, I think that in the institution itself, we are already at a level of acceptance and openness toward such people who are different in any matter, so that no one would have any problem approaching, treating or working with such a person. No importance would be given to it. He would truly be accepted as an equal. So… I think we are quite open as an institution.*—IP8). The adjective “affirmative” is in quotation marks because it is questionable if the behavior of the experts would be affirmative, that is, accepting toward persons of minority gender identities. However, in their statements, the participants are generally affirmative toward diversity. According to this attitude, hospital managers express the conviction that the availability of healthcare is not an issue and that healthcare would be provided to everyone (in the same manner) regardless of gender identity (*this cannot at any time be a reason for not helping and acting.*—IP20).

The second subtheme summarizes the expectation that healthcare team members’ reactions to transgender patients could be potentially stigmatizing. At the level of direct healthcare practice, the participants give the impression that the way in which the care was provided would depend on the individual attitudes of healthcare experts and how conservative or liberal they were in general (*I don’t know how willing our employees would actually be to even see… talk to and deal with such a person. I mean it sounds stupid. I don’t know how to put it. I don’t know, I don’t really know what staff members they would come across. Whether it would be one who has a more developed consciousness or one of those who are still more conservative*.—IP2).

Healthcare professionals might be surprised by transgender patients because they rarely meet them, so it would be unusual (*Uh, I think there would be a lot of talk about it because it’s something unknown to people, and when they meet that, such a person, I think that for everyone it’s in a way also getting to know something new, and that they would need to talk about with others. I don’t know, maybe that person would feel uncomfortable because of that.—IP4*). In addition, healthcare experts might draw conclusions about the connection between gender identity and impaired (mental) health (*if it were to be seen as part of the diagnosis, it would certainly… I strongly doubt that it would be separated from the diagnosis. One would come to the conclusion that he is like that because he is not mentally stable. I am convinced that it would be so.*—IP4).

Some also point out that they see the provision of healthcare to transgender people as a challenge in terms of not knowing enough about the medical aspects (*I believe that it would be a challenge and a question for them. So, is there a difference concerning the approach as if they were a male or female person?… So, a nose examination and ear examination are anatomical structures. I believe that the only thing, I say I don’t know either, I’m not educated about it, are these people taking some kind of permanent therapy that they have to take purely for hormonal reasons, does it affect some kind of difficulties and symptoms that cause it to occur? And that would be a challenge because you don’t treat such a person so often.*—IP1).

### 3.4. Recommendations for Improving the Healthcare of Patients with Transgender Identities

The research participants also had certain recommendations on how to improve healthcare for patients with transgender identities. Table 4 shows the subthemes related to this theme.

The participants recognize the need to “work on themselves” to be better experts in their relationships with patients with transgender identities (*That’s the same, if you as a professional don’t really like alcoholics, try to avoid them so that it doesn’t show anywhere in your professional work that you can’t really work with them because it makes you angry. Then I ask myself, well, why does it make me angry, where do I get my anger from? So, I’m always working on myself. That’s the part where we’re all weak*.—IP5). The participants also acknowledge how important the understanding of the well-being of transgender people is by experts (*if they seek help and find someone who really understands that part of the story, then I think they can definitely live more happily*.—IP5), and that it is necessary to empower patients with a transgender identity (*It actually means some kind of empowerment in the basic human dimension and for these people to see that you accept them no matter who they are. And that is actually what needs to be done the most. We are talking specifically about transgender people*.—IP5).

## 4. Discussion

Although the participants had not had much previous experience with transgender patients, they considered medical help to be available and that it would be provided regardless of their personal beliefs. In addition, they talked about the possibility of individual attitudes toward a patient having an impact on healthcare provision, depending on the personal conservativism or liberalism of healthcare team members. The participants in our research also accentuated an appreciation of diversity in their practice and the sameness of experiences with transgender patients as with other patients. However, the question of whether, due to the universal coverage of the population with healthcare and thus the theoretical accessibility of healthcare for everyone, we can consider the availability and appropriateness of healthcare still remains. According to Kcomt [32] (p. 4), acceptability refers to “professional values and norms, sociocultural factors which impact the consumer’s level of acceptance to aspects of the service, perceived appropriateness of the consumer seeking care”, while appropriateness means “the fit between the services and the consumers’ needs, the technical and interpersonal quality of the services provided”. The participants in our study connected the quality of healthcare with the attitudes of employees. Therefore, there is a need to talk about diversity competence and how to increase it. It is also interesting that a few participants rejected the possibility of the presence of discriminatory/stigmatizing behavior in their workplace. This could be a result of participants’ awareness that discriminatory behavior is something unethical and undesirable and that medical treatment needs to be provided equally to everyone regardless of personal and social characteristics, such as gender. This is prescribed at a structural level by different legislation and regulations (e.g., code of medical ethics and deontology), which is something that hospital team members are aware of. However, it is unclear whether the participants perceive everything that falls under discrimination and stigmatization, and more research is necessary in this regard in the Croatian context. Researchers already recognize that certain behaviors toward transgender individuals do not have to be a reflection of transphobia but that transgender people experience them as a reflection of stigma [33]. Kosenko et al. [33] investigated the perception of healthcare and experienced the discriminatory and stigmatizing situations of 152 transgender people in the USA. The behaviors mentioned can be divided into the following categories: gender insensitivity (e.g., use of incorrect pronouns and commenting on how well the patient “passes” as a man/woman); showing discomfort (e.g., staring and avoiding eye contact), denial of service (e.g., refusal to give an appointment); low-quality healthcare (e.g., rough examinations and ignoring pain); verbal abuse (e.g., ridicule and humiliation); forced care (e.g., imposition of certain medical procedures and psychiatric hospitalization).

The unfavorable social position of the transgender minority in society, as well as in the healthcare system, is recognized by the participants in this study, as well as in previous studies. The participants in our research perceive certain individual characteristics of patients with transgender identities and the structural factors that can influence the healthcare process. The fear of condemnation of transgender patients is recognized by the participants in this research, as well as in other studies that connect this fear with the procrastination in seeking help and the overall lower physical and mental health of transgender people [8,32,34]. They also mentioned their perception of transgender patients in terms of shame related to sexual health and an increased possibility of mental disorders in general. In a qualitative study by Stojanovski et al. [35] conducted in North Macedonia in 2021, those participants with transgender identities stated the presence of mental health problems, as well as the effect of minority stress on their identity and health. In the context of these health problems, the participants emphasized both their need for mental health services and concerns about the availability and quality of such services. They talked about stigmatization and transphobia in healthcare institutions but also acknowledged some good experiences. The participants considered healthcare professionals as “safe” if they affirmed their sexual orientation and/or gender identity [35]. Therefore, hospital staff must recognize the specific needs of transgender people, show diversity competence, and practice patient-centered care in which gender identity, sexual orientation, and other social aspects are as important as medical ones [36].

The concept of diversity competence implies the implementation of policies and practices in healthcare institutions that promote taking the individual needs of patients into account when providing healthcare, especially those who belong to various minority groups in society. Unlike the concept of cultural competence, which primarily refers to racial and ethnic minorities, the concept of diversity competence includes a much wider range of minority groups, including gender minorities [28]. The questions are, to what extent are experts in the healthcare system familiar with the concept of diversity competence? Are they aware of the need for professional development in this area? Jokić Begić, Korajlija, and Jurin [25] have already discussed transgender people in Croatia coming into contact with medical doctors and having to speak up for themselves and that doctors often lack systematic training concerning gender differences and transgenderism. The participants in this study stated that working with transgender people might be a challenge and that they need (a) more medical information—knowledge about the interaction of different medical treatments and the drugs used in treatment, and the gender reassignment process with other health conditions, and (b) an affirmative approach and self-improvement in this regard. In the context of diversity competence, educational programs should present gender identity as “a marker of minority status” [37]. The inclusion of topics related to transgender people, their health, and the health risks in the curriculum of medical studies and other professions involved in healthcare would be an important step toward improving diversity competence [10,25]. Maruca et al. have shown that experiential learning using transgender simulation might have a positive impact on affirmative practice when providing nursing care to a transgender person [38]. The education process at all levels (undergraduate, postgraduate, and lifelong learning) has the potential for the provision and gaining knowledge to help professionals in the healthcare system to better understand transgender issues, especially the needs of transgender patients from the medical perspective, but also from the broader social one.

Furthermore, it is important to note that health professionals should acquire knowledge about the social factors that affect the health of transgender patients and the social obstacles they face. Transgender people are at greater risk of social problems, such as homelessness, poverty, unemployment, social isolation, and experiencing violence, abuse, and transphobia [32,39,40]. At the same time, it is important to know that members of this population have intersecting identities, sometimes even multiple minority identities. They differ from each other in terms of age, race, ethnicity, religious beliefs, place of residence, socioeconomic status, and social background in general [6]. Such knowledge would increase the quality of co-operation with other hospital team members, such as social workers. In Croatian hospitals, health professionals contact a hospital social worker when they assess that there are social obstacles to the provision of healthcare. Besides medical treatment, it is possible to refer patients with transgender identities to a social worker for support, clinical work, and information about other resources and social services in the community.

Culturally competent practice and the influence of personal beliefs and attitudes, as well as cisnormativity, should also be addressed through formal and informal training. Training is also possible in terms of multidisciplinary co-operation between different professionals, especially in the process of lifelong learning, where other experts in the healthcare system, such as psychologists and social workers, can contribute with their knowledge. For hospital staff, focused training aimed at sensitizing employees, but also at strengthening skills in working with transgender patients, should be organized. In the Croatian context, as Jokić Begić, Korajlija, and Jurin [25] have pointed out, a transfer of knowledge from psychologists, who have gained knowledge about transgenderism, is lacking due to the hierarchical position of medical doctors above all other experts in healthcare. Therefore, it is important to research transgender people and their needs and diversity-competent practice from a different perspective. Participation in this research is also a way of improving the visibility of transgender patients and their needs in the healthcare system.

Gender minorities are also recognized as a vulnerable group from the public health perspective. A study conducted in the USA reveals that transgender people have a higher rate of suicidality compared to the general population [41,42]. If society is more inclined to stigmatize and discriminate against transgender people, it is more likely that public health interventions that include them as a target group will achieve less success. However, in Croatia, public health programs rarely even mention transgender people [19].

Due to a lack of public health institution focus, as well as the small share of transgender people in the general population [40,43], the “invisibility” of transgender people in the healthcare system is not surprising. Some participants in this research state that there was a possibility that they may have encountered a patient with a transgender identity without being aware of it. However, to reduce the health inequalities of this group of patients, it is necessary to focus on their “invisibility” [6]. An increase in visibility can be achieved at different levels. On a microlevel, it can be achieved by allowing patients to express their preferred names and personal pronouns and for these to be used when communicating with the patient. On a mezzolevel, it can be brought about by using forms that allow for genders other than those within a binary categorization, providing training for employees on diversity-competent practice and/or minority gender identities [10]. Finally, on a macrolevel, it can be achieved through public health programs aimed specifically at transgender people (e.g., enabling gynecological examinations for transgender men or advocacy for paid mastectomies through health insurance). At the same time, possible challenges and resistance to the proposed interventions need to be taken into consideration [37] and viewed through the lens of the local culture and values related to gender, gender identity, and socially expected gender roles.

In order to achieve the most satisfactory outcome, it is important that transgender people, as a targeted minority group, are involved through their associations and the engagement of activists in the creation and evaluation of public health research and interventions [41]. An interesting form of “dialogue” between experts and transgender people was applied in research by Noonan et al. [10]. Medical doctors, faculty, students, staff, lay community members, and mental healthcare providers assessed the existing situation related to the healthcare of transgender people and proposed possible improvements. It was concluded that the available healthcare was insufficiently developed and was nonaffirmative of transgender identity and individual experiences, such that the additional training of specialists was needed where members of the transgender community could participate [10]. Ultimately, according to the contemporary user perspective, members of the social group who are the focus of interventions are a very valuable source of information.

## 5. Research Limitations and Future Directions

The notable limitations of this research are the small sample and its questionable representativeness in terms of the population, the inclusion criteria, and the participant recruitment process. When taking into account the size of the healthcare system in the Republic of Croatia and the number of employees within it, it would be necessary to research a larger sample. According to the Croatian Health and Statistical Yearbook [44], 75,186 people were employed in the Croatian health system in 2021. Of the total number of employees, 20.9% are doctors, 3.1% are other health professionals and associates with a university degree, and 20.7% have a junior college education. About 63% of medical doctors are women, and 85% of the other employees are also women. If we compare the characteristics of the participants in our sample with these general data, we see that our sample is representative in terms of sex/gender (7 out of 10 participants are women). However, our sample has a higher education level than the general employee population in the healthcare system, and medical doctors are over-represented (4 participants out of 10). Furthermore, the inclusion criterion was not concerned with the previous experience of caring for patients with transgender identities but only with the experience of caring for minority patients, which is why some participants were not sufficiently informative. Nevertheless, even those participants who did not have personal experience had certain assumptions about what those experiences would be like, which reflects their attitudes.

The final recognized limitation is the selection of the health institutions/employers of the participants as gatekeepers. This factor contributed to the lower representativeness of the sample and led to the possibility that the participant’s statements were socially desirable, that is, that they had the role of “spokespersons” for their institution. In addition, it is possible that the different ways in which the interviews were conducted (face-to-face or online, with one or two interviewers) affected the collected data to a certain extent. One interviewer is female, and the other male, which is another factor that could have affected the participants’ openness. Lastly, in the invitation letter and notice facing the participants, it was stated that the researchers were employees of the Faculty of Medicine, which could have been a positive contribution to the response rate of both institutions and participants and the socially desirable answers.

Due to the small number of participants, the results of this research cannot be used for generalization purposes. However, they can guide both future research and practice. Future research should include a larger number of participants but also focus on specific professions or specializations within a particular profession. For example, we can assume that there are differences in the perception of transgender people and that experiences when caring for them vary between medical doctors, nurses, social workers, psychologists, and other professionals in the healthcare system. In addition, having previous experience in providing healthcare for transgender people should be included as an inclusion criterion, as this would result in more informative participants. Of course, over time, it would be useful to conduct research with experts from other public systems, such as the social welfare system, the education system, and others. In order to avoid “pointing fingers” at professionals regarding the possible causes of stigmatized practices, future research should uncover the underlying causes, such as social values, structural constraints, and challenges in the provision of healthcare, both overall and toward minority groups. In general, it would be useful to examine the preliminary findings obtained through qualitative research such as this on a larger survey research sample and vice versa [11].

## 6. Conclusions

This research aimed to gain insight into the perception of the provision of healthcare services to patients with transgender identities by healthcare managers and hospital team members in the healthcare system in the Republic of Croatia. The results of this research show that only some of the participants had had an encounter with patients with transgender identities. An equal number of participants imagine future encounters as either affirmative or potentially stigmatizing. Most of all, they emphasize the possibility of being surprised by a “different” patient. Participants believe that healthcare is available to transgender patients and that medical help would be provided regardless of personal beliefs and attitudes. The participants also recognized the necessity of the self-improvement of experts and an affirmative approach as a means of improving the quality of healthcare for transgender people. We identified the training of hospital staff and dialogue between patients and experts as additional ways of achieving these goals. We also recognize that additional research is needed to supplement the knowledge about the experiences of transgender people in the Croatian healthcare system.

## Figures and Tables

**Table 1:** Experiences with the provision of healthcare to transgender patients.

Theme	Subthemes
Past experiences of the provision of healthcare to transgender patients	Lack of experience in providing healthcare to transgender patients
	Past experiences of provision of healthcare to transgender patients
	The sameness of experiences with transgender patients as with other patients

**Table 2:** Perception of patients with transgender identities.

Theme	Subthemes
Perception of patients with transgender identities	Perception of individual characteristics of patients with transgender identities
	Perception of factors in the environment of patients with transgender identities

**Table 3:** Assumed reactions of hospital staff to patients with transgender identities.

Theme	Subthemes
Assumed reactions of hospital staff to patients with transgender identities	“Affirmative” reactions
	Potentially stigmatizing reactions

**Table 4:** Recommendations for improving the healthcare of patients with transgender identities.

Theme	Subthemes
Recommendations for improving the healthcare of patients with transgender identities	The need for the self-improvement of hospital team members
	The need for an affirmative approach

## Data Availability

All data included in the analysis during this study are available on request from the corresponding author.

## Appendix A. Interview Guide

1. Have you had experience with transgender people during your work? Please, recall that experience/event. Describe it. (Recall some, for you, pleasant experiences/some unpleasant experiences).

How did you behave then? Are you satisfied with the way you behaved? What would you do differently now?

2. How often do you meet transgender people? Why do they turn to you? what do they need, and what are their needs?

3. If there have been no experiences before—If you had a transgender person as a patient, how would you behave? What would be a challenge for you?
